# Differential CT and MR imaging diagnosis between low- and high-grade malignant vascular tumors of bone

**DOI:** 10.1186/s40064-016-3471-z

**Published:** 2016-10-12

**Authors:** Jian-Xia Xu, Li Yang, Ying Chen, Mohamad Wasil Peeroo, Xiu-Liang Zhu, Yan-Biao Fu, Ri-Sheng Yu

**Affiliations:** 1 Department of Radiology, The Second Affiliated Hospital, Zhejiang University School of Medicine, No. 88 Jiefang Road, Hangzhou, 310009 Zhejiang China; 2 Department of Radiology, Shenzhen People’s Hospital, The Second Clinical Medical College, Jinan University, No. 1017 Dongmen North Road, Shenzhen, 518020 Guangdong China; 3 Zhejiang University School of Medicine, No. 866 Yuhangtang Road, Hangzhou, 310058 Zhejiang China; 4 Department of Pathology, The Second Affiliated Hospital, Zhejiang University School of Medicine, No. 88 Jiefang Road, Hangzhou, 310009 Zhejiang China

**Keywords:** Malignant vascular tumors of bone, Low-grade, High-grade, CT, MR imaging, Differential diagnosis

## Abstract

**Objective:**

To investigate the CT and MR imaging findings and differential diagnosis of malignant vascular tumors of bone.

**Materials and methods:**

CT and MR imaging findings of 18 patients with histopathology-proven malignant vascular tumors of bone were examined. Assessed image features included age, sex, location, CT findings, and MR imaging appearances and dynamic contrast-enhanced MR imaging.

**Results:**

The study group comprised 18 cases, of which 7 were categorized as low-grade malignant vascular tumors (LMT), and 11 were categorized as high-grade malignant vascular tumors (HMT). Malignant vascular tumors of bone showed osteolytic lesions with heterogeneous signs and enhancement, and frequently associated with soft tissue masses and peritumorous edema. The mean age of patient was respectively 34.7 years in LMT with 3 patients younger than 20 and 49.9 years in HMT with 3 patients older than 60 years. The mean lesion diameter was 3.6 cm (range 2–7.2) in LMT with two lesions <3 cm and 7.1 cm (range 3–13) in HMT with 3 lesions greater than 10 cm. LMT showed multifocal (57.1 %) and well-defined (71.5 %) lesions with residual bone (57.1 %), peripheral sclerosis (85.7 %), and slightly hetergeneous enhancement (71.4 %), compared to those of HMT with 9.1, 45.5, 27.3, and 72.7 %, and 9.1 % respectively. Also, HMT appeared as expansive (54.5 %), ill-defined (54.5 %), macroscopic necrosis/cystic (81.8 %) or hemorrhagic (27.3 %) lesion with pathological fracture (27.3 %), and often presented with obviously hetergeneous enhancement (81.8 %), compared to those of LMT with 42.9, 28.6, 42.9, 0, 14.3 and 14.3 % respectively.

**Conclusions:**

There are some differences in the imaging features between LMT and HMT, while unifocal/multifocal, expansive, ill-defined, necrosis/cystic, hemorrhagic features with age, lesion diameter, peripheral sclerosis, residual bone tissue, pathological fracture and slightly/obviously hetergeneous enhancement highly suggest their differential diagnosis.

## Background

In the 2002 World Health Organization (WHO) classification, vascular bone tumors were formerly categorized as hemangiomas or angiosarcomas (AS), with endothelioma being subsumed by the latter (Fletcher 2006). The WHO classification of bone and soft tissue tumors has been updated in 2013, where a new type of vascular tumor, the epithelioid sarcoma-like hemangioendothelioma (ES-H) is newly described (Jo and Fletcher 2014). Malignant vascular tumors of bone (MVTB) are exceedingly rare representing <1 % of primary malignant bone tumors and are categorized as hemangioendothelioma (HE), AS and their epithelioid variants. Accurate preoperative diagnosis of these tumors is very difficult because they may mimic both indolent, benign lesions and metastases (Wenger and Wold 2000a, b).

Most published series of MVTB include both low-grade malignant vascular tumors (LMT) and high-grade malignant vascular tumors (HMT). HE, ES-H and epithelioid hemangioendothelioma (EHE) are usually considered LMT, whereas AS and epithelioid angiosarcoma (EA) are regarded as HMT. Because the biologic behavior of MVTB is also widely variable depending largely upon the grade of the tumor, it is important to effectively and accurately distinguish them from each other. Some imaging findings in vascular tumors of bone have been reported (Wenger and Wold 2000a, b; Vermaat et al. 2011; Errani et al. 2012; Griffith et al. 2013). To the best of our knowledge, there are no reports in the English literature describing the differences of imaging features between LMT and HMT. The radiological appearance of MVTB is non-specific (Vermaat et al. 2011), and these tumors are often grouped together for analysis owing to their similar radiological findings.

Our aim was to review the clinical and imaging features of a series of proven MVTB and determine the differential diagnosis between LMT and HMT.

## Methods

This retrospective study was approved by the institutional ethical review board, and an informed consent was waived. We reviewed the medical records of 18 patients with histopathology-proven MVTB at our hospital from January 2010 to August 2015. Of these 18 cases, 2 were categorized as HE, 4 as EHE, one as ES-H, 9 as AS, and 2 as EA. The clinical information was extracted from the medical records.

CT scanning was performed with 16- or 64-row multidetector CT scanner (Somatom Sensation, Siemens Medical Systems). MR scanning was performed with a 1.5 T or 3.0 T magnets (Signa, GE Medical Systems).

Among the 18 patients, CT scan was performed in 18 patients and MR imaging in 16 patients. Sixteen patients underwent contrast enhanced MR scans and 4 patients underwent contrast enhanced CT scans.

In each patient the imaging findings were evaluated by two experienced radiologists (each with more than 20 years of experience in musculoskeletal tumors) and the features were recorded by consensus. For all cases, the following information about imaging findings were noted by the radiologist: age, sex, location, size (the largest diameter), matrix (lytic, sclerotic, lytic-sclerotic), multifocality, expansion, presence of residual bone, margin, cortex, peripheral sclerosis, periosteal reaction, pathologic fracture, signal intensity of images on T1- and T2-weighted sequences compared to the intensity of the surrounding muscle, presence of macroscopic necrotic or cystic component (defined as pronounced hyperintense signal on T2-weighted images), presence of macroscopic hemorrhagic component (defined as focal T1-weighted hyperintense area), peritumoral signal intensity, intensity of enhancement.

## Results

### Clinical information

The clinical features are summarized in Table 1. The 7 patients with LMT ranged from 18 to 59 years old (4 M, 3 F; mean age 34.7 years) with 3 patients younger than 20 years. The 11 patients with HMT ranged from 26 to 82 years (7 M, 4 W; mean age 49.9 years) with 3 patients older than 60 years. The long bones in the lower extremity were predominantly affected in 3 cases (46.2 %) with LMT and 4 cases (36.4 %) with HMT. Four cases (36.4 %) of AS occurring in the vertebrae were seen. The mean lesion diameter was 3.6 cm (range 2–7.2) in LMT with 2 lesions <3.0 cm and 7.1 cm (range 3–13) in HMT with 3 lesions greater than 10 cm. Patients presented with localized pain (n = 13), soreness (n = 4), numbness (n = 2), motor weakness and sensory abnormality (n = 1), swelling (n = 1).

**Table 1:** Clinical information

Case	Diagnosis	Age (year)/sex	Location 15	Size (cm)	Clinical presentations
1	HE	18/F	Left mandible	2	Pain and numbness
2	HE	58/M	T8 vertebral body	5	Motor weakness and sensory abnormality
3	ES-H	18/F	Proximal metacarpal	2.7	Soreness
4	EHE	18/M	Distal femur, proximal tibia	4	Pain
5	EHE	19/M	Distal femur, proximal tibia, proximal humerus, scapula	4.8	Pain
6	EHE	50/M	Distal femur	4.5	Pain
7	EHE	59/F	Clavicular acromial end, scapula	7.2	Pain
8	AS	26/M	Femoral neck	6.8	Pain
9	AS	37/F	Proximal tibia	3	Pain
10	AS	42/F	Proximal humerus	9	Soreness
11	AS	42/F	Mid humerus	4	Pain
12	AS	47/M	Sacrum, ilium, L5 vertebra	13	Pain; numbness
13	AS	50/M	L2 vertebral body and pedicle of vertebral arch	4.6	Pain
14	AS	58/M	Pelvis, L5 vertebra involved	12	Pain
15	AS	75/M	T8 vertebral body	5	Pain
16	AS	82/M	Proximal humerus	6	Soreness
17	EA	28/F	Femoral neck	4	Swelling; soreness
18	EA	62/M	Femoral neck	10.8	Pain

### Imaging findings

The imaging findings of all cases were summarized in Table 2 and statistical differences between LMT and HMT in Table 3, details of which were as follows.

**Table 2:** Summary of imaging features in 18 cases

Case	Radiological features		Diagnosis
	CT	MRI	
1	Purely lytic	No MRI obtained	HE
2	Lytic, lattice-like trabeculation, cortical destruction, compression, paraspinal mass, artery passed through the lesion without involvement	No MRI obtained	HE
3	Lytic	Hypo/isointense on T1, hyperintense on T2, heterogeneous enhancement with strong enhancement	ES-H
4–6	Lytic, multiple, lobulated, residual bone	Heterogeneous hypointense on T1, hyperintense on T2, periotumoral hypointense rim	EHE
7	Lytic, multiple, geographic	Ill-defined, cystic/necrotic, infiltrative, heterogeneous hypointense on T1, hyperintense on T2, slightly heterogeneous enhancement	EHE
8, 10, 15	Lytic	Hypointense on T1, slightly heterogeneous hyperintense on T2, obviously heterogeneous enhancement	AS
9	Lytic, pathologic fracture	Ill-defined, hypointense on T1,hyperintense on T2, homogeneous enhancement	AS
11, 13, 16	Lytic	Ill-defined, mixed hypo-/hyperintense on T1 and T2, massive shape, obviously heterogeneous enhancement	AS
12	Lytic, multiple, residual bone	Hypointense on T1, slightly hypo/hyperintense on T2, slightly heterogeneous enhancement	AS
14	Lytic-slerotic, large soft-tissue mass, residual bone	Markedly hypo/hyperintense on T1 and T2, macroscopic cystic/necrosis, obviously heterogeneous enhancement	AS
17	Lytic	Ill-defined, isointense on T1, iso/hyperintense on T2, obviously heterogeneous enhancement	EA
18	Lytic, pathologic fracture	Ill-defined, hypo/hyperintense on T1 and T2, hemorrhage, obviously heterogeneous enhancement	EA

Similar imaging features were merged in one table

**Table 3:** Statistical differences in the imaging features between LMT and HMT

	LMT (N = 7)				HMT (N = 11)		
	HE (n = 2)	ES-H (n = 1)	EHE (n = 4)	P^a^ (%)	AS (n = 9)	EA (n = 2)	P^a^ (%)
Multifocality	0	0	4	57.1	1	0	9.1
Expansion	0	1	2	42.9	5	1	54.5
Residual bone	1	0	3	57.1	3	0	27.3
Peripheral sclerosis	1	1	4	85.7	6	2	72.7
Cortical destruction	1	1	4	85.7	8	1	81.8
Periosteal reaction	0	0	0	0	0	0	0
Pathologic fracture	1	0	0	14.3	2	1	27.3
Cystic/necrotic component	NA^b^	1	2	42.9	7	2	81.8
Hemorrhage	NA^b^	0	0	0	2	1	27.3
Ill-defined margin	1	0	1	28.6	5	1	54.5
Enhancement							
Homogeneous	0	0	0	0	1	0	9.1
Slightly heterogeneous	1	0	4	71.4	1	0	9.1
Obviously heterogeneous	0	1	0	14.3	7	2	81.8

^a^Proportion; ^b^ not available

**Fig. 1:**
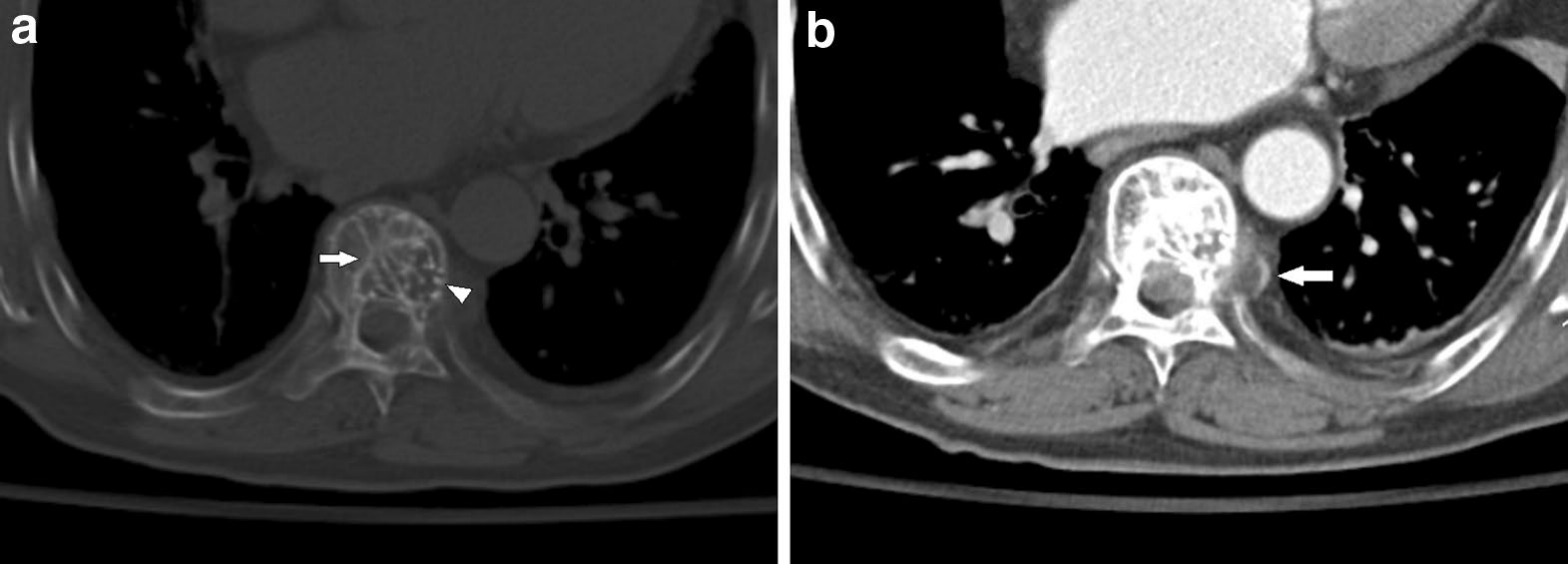
Case 2: **a** Unenhanced CT image at bone window settings shows that a lytic lesion with lattice-like coarse trabeculae, interrupted cortex (*arrowhead*), and partial peripheral sclerosis (*white arrow*). **b** Enhanced CT image shows paraspinal mass and posterior intercostal artery (*white arrow*) passing through the lesion without involvement

One of 2 cases with HE showed mild compression of the T8 vertebral body, with lattice-like coarse trabecular pattern (Fig. 1a). In enhanced CT, the lesion showed marked enhancement and infiltrated the surrounding soft tissue forming paraspinal mass. The posterior intercostal artery passed through the lesion without involvement (Fig. 1b).

**Fig. 2:**
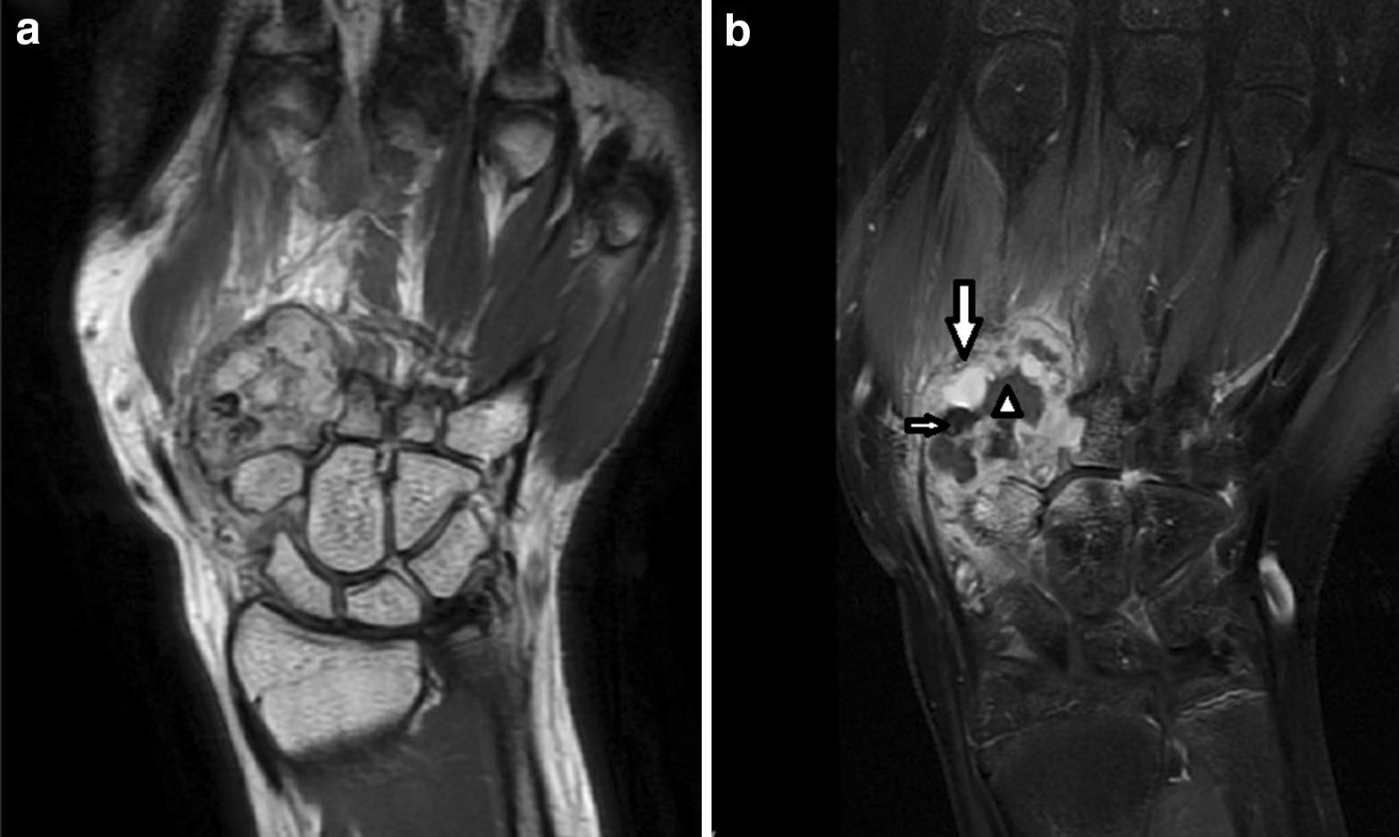
Case 3: **a** Coronal T2-weighted MR image shows a relatively well-defined mass with heterogeneous hyperintensity on T2WI. **b** Coronal fat-supressed T1-weighted postcontrast image shows cystic/necrotic components and thrombus (*short arrow*) inside the lesion with very strong enhancement (*long arrow*) of tumor tissue within the lesion and enhancement in the surrounding soft tissue

One case with ES-H showed lytic, expansive, relatively well-defined lesion with cortical destruction. The lesion also showed cystic/necrotic components and thrombus inside the lesion (Fig. 2a, b). The contrast enhanced T1WI showed very strong enhancement of tumor tissue within the lesion (Fig. 2b). Ill-defined high signal on T2WI and enhancement on T1WI located in the surrounding soft tissue were seen.

**Fig. 3:**
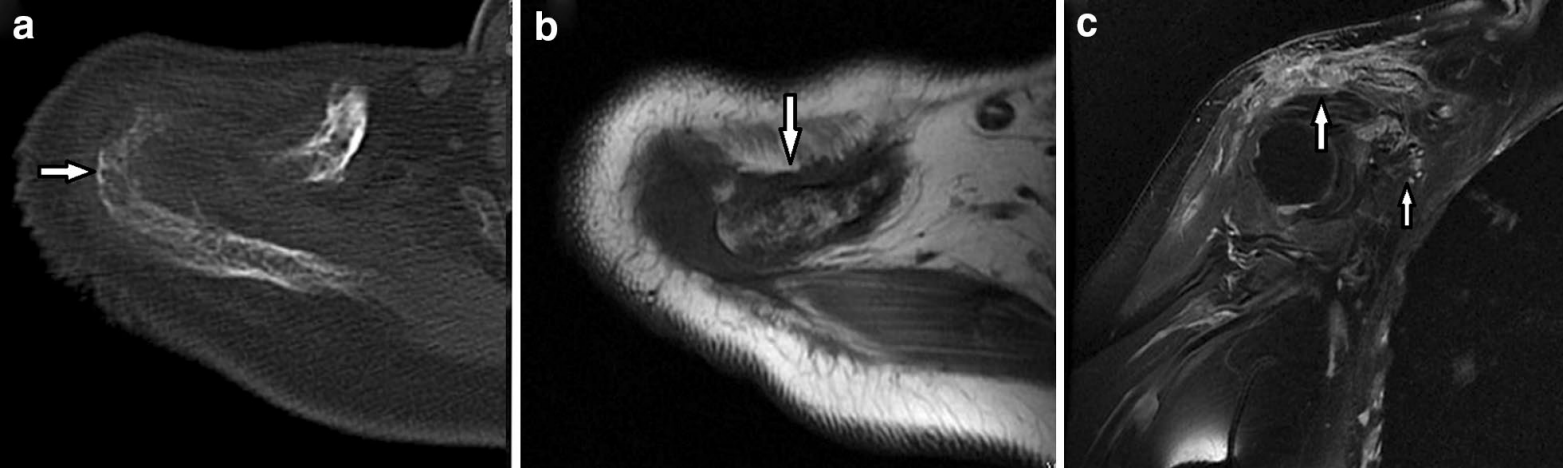
Case 7: **a** Unenhanced CT image shows irregularly bone destruction (*white arrow*). **b**, **c** Axial T1-weighted MR image and coronal fat-supressed T1-weighted postcontrast image show multiple, ill-defined lesions of the acromioclavicular joints and coracoid (*white arrow*) with geographically pattern and heterogeneous enhancement

Three cases with EHE showed multifocal, oval or lobulated, mildly expansive, and lytic lesions with peripheral sclerosis, partial cortical disruption and residual bone. The other one case manifested as aggressive behavior, showing multifocal, lytic, ill-defined and permeative, with a geographically pattern, disrupted cortex and residual bone (Fig. 3a–c).

**Fig. 4:**
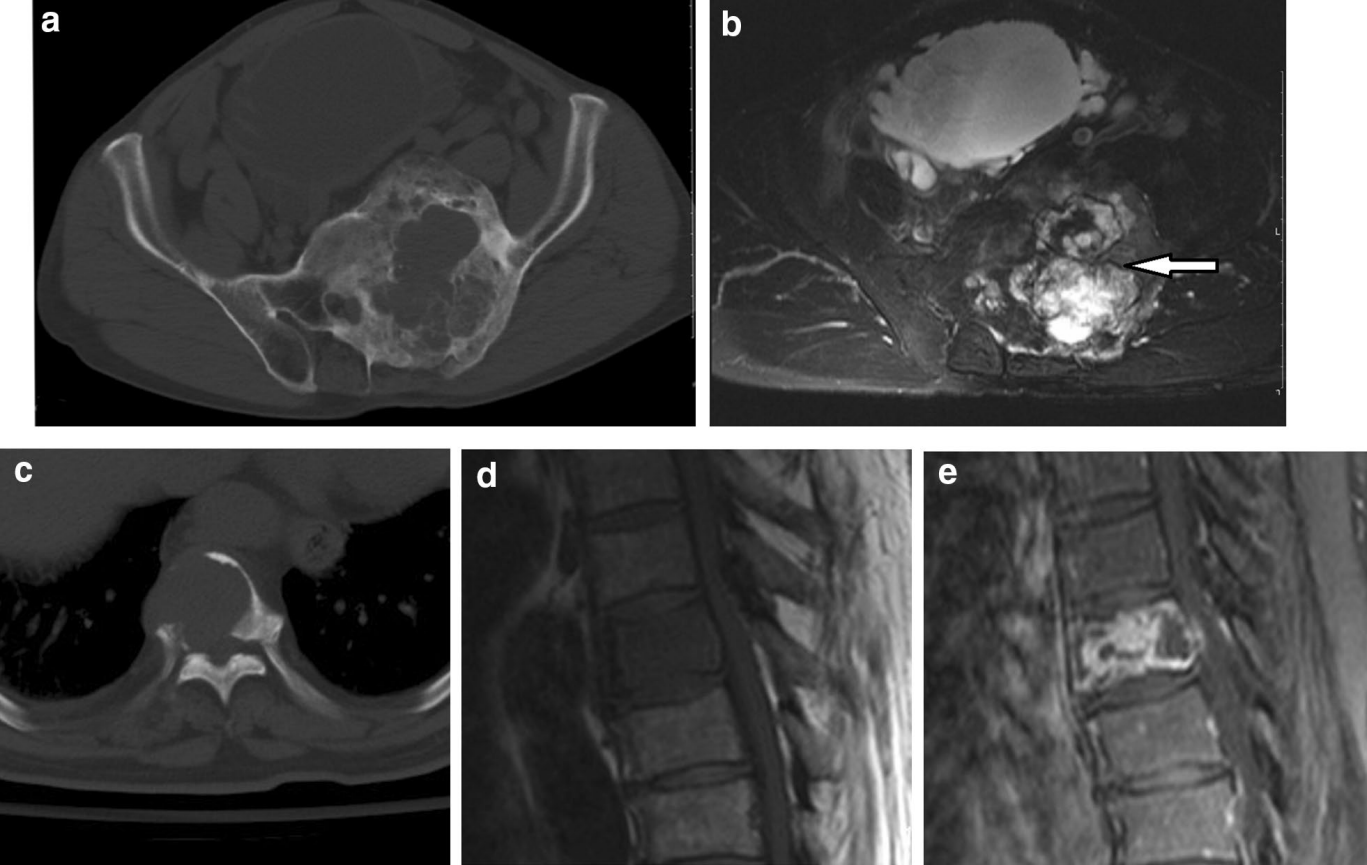
Case 14–15: Case 14, **a** Axial CT image demonstrates a large mixed lytic-sclerotic lesion, with a massive soft tissue mass confined to bone. **b** Axial fat-supressed T2-weighted MR image shows a high hetergeneous signal indicating cystic/necrosis components (*white arrow*) inside the lesion. Case 15, **c** Unenhanced CT image at bone window settings shows bone destruction with purely lytic appearance with discontinuous cortex and paraspinal mass. **d**, **e** Sagittal T1-weighted image and fat-suppressed T1-weighted postcontrast image show a heterogeneous hyperintensity and mild compression of the vertebral body

Radiographs of AS showed lytic-sclerotic lesion in one case and lytic lesions in 8 cases (Fig. 4a–c). MRI showed mixed signal intensity on T1WI and T2WI, with macroscopic cystic/necrosis components inside the lesions in 7 cases and hemorrhage in 2 cases (Fig. 4b). The contrast enhanced T1WI showed heterogeneous enhancement (Fig. 4d–e). Ill-defined high signal on T2WI and enhancement on T1WI located in the surrounding bone were seen in one case.

**Fig. 5:**
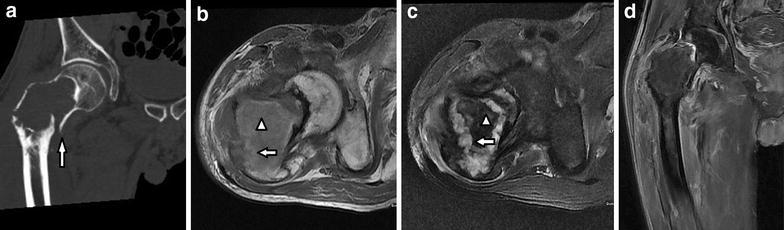
Case 18: **a** Coronal CT image shows a pathological fracture (*white arrow*) of the neck of femur secondary to an osteolytic destructive lesion with soft-tissue mass. **b**, **c** Axial T1-weighted and fat-supressed T2-weighted MR images show a large heterogeneous mass and hypointense and hyperintense on T2WI in the centre of the lesion are suggestive of hemorrhage (*arrowhead*) and necrosis (*white arrow*) respectively. **d** Coronal fat-supressed T1-weighted postcontrast image shows that the lesion has an enhancement in the surrounding soft tissue

One of 2 cases with EA showed cortical destruction, pathologic fracture, and invasion of the tumor to the surrounding tissues with extensive hemorrhage and necrosis in central area (Fig. 5a–c). Ill-defined high signal on T2WI and enhancement on T1WI located in the surrounding bone were also seen (Fig. 5d).

## Discussion

Pathologically, some vascular tumors described above are remarkably similar, which makes differentiating them from each other very difficult. Most published series of MVTB include low-grade and high-grade lesions. Errani et al. (2012) described AS and EA as HMT, while they considered HE and EHE as LMT, although EHE also had intermediate malignancy potential. Another type of intermediate vascular tumor, termed ES-H, was newly described in 2013 (Jo and Fletcher 2014) and Steven et al. (Billings et al. 2003) considered it as LMT. Therefore, in our study, we described HE, ES-H and EHE as LMT, AS and EA as HMT.

HMT is more likely to occur in elderly patient compared with LMT and there was no sex predilection in LMT but a slight male predominance existed in HMT (Errani et al. 2012). As a matter of fact, in a review of 50 ES-H cases, Hornick and Fletcher found 41 (82 %) patients were male (Hornick and Fletcher 2011). According to the present 21 cases, we believe that being a younger patient, especially <20 years old, and small in lesion diameter (<3 cm), was a contribution to the diagnosis of LMT, and for the elderly patients, especially being older than 60 years old and larger in lesion diameter (>10 cm) might be suggestive of HMT. It is not mentioned in the previous literature. Both LMT and HMT predominantly affect the long bones of the lower extremity (Vermaat et al. 2011; Wold et al. 1982; Amary et al. 2013; Fayad et al. 2006; Larochelle et al. 2006; Verbeke et al. 2011; Peacock et al. 2013). HE is relatively common in the tibia, femur and humerus. By contrast, all 2 cases in our study occur in the mandible and vertebral. Four cases with AS (36.4 %) occurred in the vertebrae, which may also be its predilection site. When the case is multifocal, it has a tendency to involve a single anatomic region or the extremities in ES-H and EHE, and one or more anatomical regions in AS (Wenger and Wold 2000a, b; Boutin et al. 1996). Clinical presentations of patients in our study are mainly painful (95.2 %) and may be associated with pathological fractures.

The imaging findings of MVTB have been reported in several previous literatures, and generally, it was considered very challenging to differentiate them from each other. According to the present 18 cases, MVTB usually reveal single or multifocal, osteolytic, and variably expansile lesions with heterogeneous signs and marked enhancement, and are frequently associated with soft tissue masses, without periosteal reaction. In our study, there was also one case of AS showing mixed lytic and sclerotic pattern. The reactive changes were seen in our series as ill-defined high signal on T2WI and enhancement on T1WI located in the surrounding bone and/or soft tissues, which may reflect the presence of reactive edema, neovascularization or diffuse tumor extension (Wenger and Wold 2000a, b). Nonetheless, we also noted some differences among the subtypes of MVTB in our study. Partial cases in our study pathologically confirmed negative margin, so we believed that it tends to be reactive oedema.

Besides, we found that LMT tends to have multifocal and well-defined lesions with residual bone tissue, peripheral sclerosis, and slightly hetergeneous enhancement, whereas HMT is more likely to be associated expansive, ill-defined, necrosis/cystic, hemorrhagic, pathologic fracture and often presents with obviously hetergeneous enhancement. The incidence of expansion, peripheral sclerosis, disrupted cortex, pathological fracture, and homogeneous enhancement in LMT and HMT was almost similar. These similar or different imaging features of LMT and HMT were not summarized in the previous literatures.

Although HE typically presents radiographically as single or multiple lesions with a lytic pattern of bone destruction, they may additionally present as mixed lytic and sclerotic lesions (Vermaat et al. 2011; Errani et al. 2012; Baliaka et al. 2013). The imaging findings in our study were similar to typical appearances. Furthermore, one patient with HE from our cases demonstrated both benign and malignant features, probably because of its low-grade malignancy, and this characteristic might be helpful to make the diagnosis. To the best of our knowledge, there has been no relevant literature reported previously. When the lesion featuring this appearance occurs in the vertebrae, HE should be entertained as a diagnosis. Lesions of EHE are characteristically multifocal, oval, well circumscribed, with marked peripheral sclerosis, and located around the knee, which are highly suggestive of the diagnosis (Boutin et al. 1996). EHE can also occur in other location simultaneously, but is rarely reported. We reported the case as having aggressive lesions centered in the medullary cavity but with extension through the cortex and into the surrounding soft tissue, which could occasionally be identified in the previous studies (Boutin et al. 1996; Weissferdt and Moran 2014). Such a case can often be quite complex and difficult to diagnose. Currently, there is little literature regarding ES-H of bone. Primary bone lesions may be aggressive, with cortical destruction and soft tissue invasion (Karakasli et al. 2014; Xu et al. 2012). ES-H in our study showed cortical destruction, marked cystic/necrotic component, and abnormally enhanced tumor tissue, which was suggestive of malignant vascular tumor of bone. According to our series, lytic lesions with marked enhancement, especially involving the vertebrae, are highly suggestive of AS. EA of bone is extremely rare and the literature is limited to only several case reports (Errani et al. 2012). This finding that heterogeneous sign with extensive hemorrhage in our case was seen is in agreement with a report that extensive hemorrhage, necrosis, and cystic degeneration were present in EA, which we believed that extensive hemorrhage was the predominant feature of the tumor (Chen et al. 2011). Whether these features are helpful to confirm the diagnosis of the disease requires further studies.

This study has several limitations. Firstly, the patient population in our study is relatively small with limited statistical power. Secondly, the lack of uniform imaging modalities and scan protocols were also secondary to the retrospective nature of this study.

In conclusion, there are some differences in the imaging features between LMT and HMT, while unifocal/multifocal, expansive, ill-defined, necrosis/cystic, hemorrhagic features with age, lesion diameter, peripheral sclerosis, residual bone tissue, pathological fracture and slightly/obviously hetergeneous enhancement highly suggest their differential diagnosis.

## Abbreviations

LMT: low-grade malignant vascular tumors
HMT: high-grade malignant vascular tumors
MVTB: malignant vascular tumors of bone
HE: hemangioendothelioma
EHE: epithelioid hemangioendothelioma
ES-H: epithelioid sarcoma-like hemangioendothelioma
AS: angiosarcomas
EA: epithelioid angiosarcoma
